# Common Errors in Digital Panoramic Radiographs of Patients with Mixed Dentition and Patients with Permanent Dentition

**DOI:** 10.1155/2012/584138

**Published:** 2012-02-08

**Authors:** Benjamin Peretz, Maya Gotler, Israel Kaffe

**Affiliations:** ^1^Department of Pediatric Dentistry, The Maurice and Gabriela Goldschleger School of Dental Medicine, Tel Aviv University, Tel Aviv 69978, Israel; ^2^Department of Oral Pathology and Oral Medicine, The Maurice and Gabriela Goldschleger School of Dental Medicine, Tel Aviv University, Tel Aviv 69978, Israel

## Abstract

*Purpose*. To compare errors in digital panoramic radiographs of permanent and mixed dentitions. *Methods*. 143 and 146 digital radiographs of mixed and permanent dentitions were examined. *Results*. Significantly fewer errors presented in the mixed dentition. Positioning too forward significantly prevalent in the mixed dentition; slumped position and nonpositioning of chin properly were significantly prevailed in the permanent dentition. Blurred or shortened upper incisors were significantly more prevalent in the mixed dentition. Diagnostic ability could be improved by manipulating the brightness or contrast in nearly 45% of all radiographs. In the mixed dentition, tilting the chin down and a slumped position made the lower incisors significantly nondiagnostic. In the permanent dentition, tilting the chin down made the lower incisors to be significantly nondiagnostic. *Conclusions*. More errors were prevalent in panoramic radiographs of permanent dentitions. Properly positioning the patient is the most important factor in preventing a cascade of errors.

## 1. Introduction

Panoramic radiographs have long been one of the most common means for imaging dental structures among dentists due to their many advantages [1, 2]. A panoramic radiograph provides an overview of both dental arches and a close view of a large number of anatomical structures such as the maxillary sinuses, the temporomandibular joint (TMJ), and the hyoid bone. In addition, it is simple to take, and can be carried out in patients whose mouth opening is limited [3–5].

However, the panoramic radiograph also bears some disadvantages. It provides less sharp images and less accurate information about dental and oral diseases than regular intraoral periapical or bite-wing radiographs [6–8].

Another disadvantage of the panoramic radiograph is the distorted picture that is very often seen [9–11]. Furthermore, panoramic radiographs may contain radio-opaque and radiolucent spots that are reflections of various structures on the examined areas as well as shadows of soft tissues and anatomical air spaces [3, 4, 12, 13]. The dentist must be familiar with these areas in order to accurately interpret the radiograph.

The quality of any radiograph depends on accurate technique and careful processing of the image. Correct positioning of the patient is essential for a sharp, accurate, and undistorted image, which is not affected by ghost images. In addition, quality control is crucial when interpreting the image [14–16].

The following technical points are important when a panoramic radiograph is taken [17].

1. The patient should be seated or should stand fully upright, with the head immobilized, utilizing a chin rest and a radiolucent bite block.
2. Spectacles, neck chains, earrings, and dentures must be removed before the exposure.
3. The patient should place the tongue against the palate during the exposure in order to prevent a radiolucent stripe above the maxillary teeth.
4. Because of the relatively long exposure, the machine movement should be explained to the patient to ensure cooperation (especially relevant with children).

A number of studies examined the quality of panoramic radiographs and the frequency of mistakes when interpreting them. Schiff et al. [5] examined 1000 panoramic radiographs taken in a dental school by students, faculty members, and X-ray technicians. The authors concluded that 80% of the radiographs taken by dental students and faculty members contained errors in patient positioning or in the processing of the radiographs themselves. It was demonstrated that skilled X-ray technicians were able to produce an error-free radiograph in only 47% of the radiographs. Brezden and Brooks [15] examined 500 radiographs and found only one error-free radiograph. The average radiograph contained 4.7 distortions. Rushton et al. [16] investigated the quality of 1813 panoramic radiographs of patients aged 18 years or older in private clinics and found that only 0.8% of the radiograph were totally suitable for accurate diagnoses, 66.2% were marked as accepted and 33% were totally unaccepted for diagnoses. A recent study which evaluated the frequency of errors in panoramic radiographs in young orthodontic patients and registered pathologic and abnormal conditions found that 96% had errors. The number of errors in each image varied from 1 to 5 [18].

The dental literature contains no studies on comparisons of the quality of panoramic radiographs and their diagnostic ability between young patients with primary or mixed dentition and young adults with permanent teeth. It is logical to assume that panoramic radiographs of young patients with mixed dentition will contain more faults in patient positioning or movement during the radiograph than radiograph of older patients with permanent dentition [16].

This is important since many panoramic radiographs are taken for orthodontic purposes and contain mixed dentitions.

Therefore, the purpose of the present study was to compare the errors in panoramic radiographs of permanent and mixed dentitions.

## 2. Materials and Methods

For this study, 289 digital panoramic radiographs of mixed and permanent dentitions, which were taken for clinical purposes at the Institute of Radiology of Tel Aviv University in Tel Aviv, were evaluated after approval by the University's Ethical Committee.

All panoramic radiographs of the patients with mixed and permanent dentitions that have been taken from December, 2008 to December, 2009 were examined. Radiographs of edentulous patients were excluded. There were 143 radiographs of patients with mixed dentition, and 146 radiographs of patients with permanent dentition. The age range of the patients was from 10 years to 55 years.

All radiographs were carried out by the same technician with the same equipment (Kodak 8000c Digital Panoramic and Cephalometric System Carestream Health Inc., Rochester NY, USA), operating at 70 Kilovolts and 10 milliamperes. Exposure time was 13 seconds. Images were processed using Kodak Dental Software.

The radiographs were reviewed under identical conditions, on a computer screen (Lenovo T510 laptop with 15-inch screen, 1600 × 900 screen resolution, 32 bit color mode), in a room with subdued lighting. One experienced dentist evaluated the radiographs after evaluating with the other coauthors 10 radiographs were not included in this study, according to criteria described by Langland and others [3, 4].

**Patient Positioning Errors**

1. *Patient is positioned too far forward*. The anterior teeth are narrowed, sometimes with pseudospaces. The crowns of these teeth may appear fractured because they are cut out of the image layer. The cervical spine is superimposed symmetrically on both sides on the ramus and condyles. Overall, the whole image appears to have been narrowed.
2. *Patient is positioned too far backward*. The anterior teeth are widened. The lower turbinate and meati are spread-out bilaterally across the maxillary sinus. The condyles appear to be almost off the lateral edges of the image. Ghost images of the contralateral rami are superimposed symmetrically and bilaterally on the posterior molars and rami. Overall, the image appears too large.
3. *The patient's head is twisted or turned*. The posterior teeth on one side are widened and overlap interproximally. On the other side, the posterior teeth are narrowed. On the side with the wide posterior teeth, the ramus is widened; on the other side, the ramus is narrowed and intersected by a ghost image of the contralateral ramus. On the side with the wide ramus, the inferior turbinate and meati are spread out across the maxillary sinus.
4. *The patient's head is tilted in the machine.* The posterior teeth may be widened on one side with a widening gap between the upper and lower teeth; on the other side, the interproximal gap is narrowed. The lower edge of the mandible on one side is almost horizontal; on the side with the widened interocclusal gap, the mandible is enlarged and the lower edge appears to be directed upward above the horizontal plane. Also, on this side, the condyle is enlarged and is above the contralateral condyle, which is smaller and lower in the image.
5. *The chin is tipped too low*. The “smile” line created by the interocclusal gap is exaggerated. The apices of the mandibular anterior teeth are “cut-off.” The mandible is widened vertically on the anterior region, with poor imaging of the trabecular pattern. The mandible is transversed by an elongated double image or ghost of the hyoid bone. The condyles approach the upper edge of the image or are cutoff by its upper edge.
6. *The chin is raised too high*. The “smile” line is lost entirely; the occlusal plane appears flat or even in a reverse curve or “sad” configuration. The real, double, and ghost images of the palate form a widened, prominent, radiopaque line, which is projected downward to approximate or superimpose on the apices of the maxillary teeth. The condyles approach the lateral edges of the image or are projected off its edges symmetrically and bilaterally.
7. *The patient is slumped*. The patient's neck is stretched forward on a slant, causing a ghost image to be produced in the middle of the image. Thus, the anterior teeth are difficult to see, as the ghost radiopaque image of the spine has superimposed on this area and obliterated it.
8. *The chin is not positioned on the chin rest*. The zones of the nosesinuses are “cutoff” by the upper edge of the image. Its lower edge corresponds with the lower edge of the chin rest, creating a large distance from the lower edge of the image.
9. *The tongue is not on the plate or the lips are open*. The crowns of the upper and lower teeth are obscured by the air between the parted lips. The apical region of the maxillary teeth is obscured by dark air space between the dorsum of the tongue and the hard and soft palates (palatoglossal air spaces).
10. *Bite guide is not used*. Incisal and occlusal surfaces of the upper and lower teeth overlap.
11. *The patient moves*. Along the inferior cortex of the mandible, an interruption in its continuity can be seen, especially in the molar area.

**Technical Errors**

1. *The patients wear prostheses or jewelry*. Radiopaque projections of prostheses or jewelry may be observed on the radiograph.
2. *Incorrect starting point (home base)*. A portion of the film is black. A portion of the anatomy is lost at the edge of the film.
3. *Apron/thyroid shield artifact*. Using an intraoral style of apron or thyroid shield instead of the “poncho” style will result in projections onto the radiographs as blank or clear underexposed (opaque) areas.

Errors associated with film radiographs, such as static electricity or exposure problems, were not examined in our study. Our study focused on digital panoramic radiographs that did not need to insert and remove a film to the cassette, and exposure problems can easily be dealt by changing brightness, sharpness, and shades. Every radiograph may contain a number of errors.

Besides examining the errors, the diagnostic power of each radiograph was established. A radiograph was categorized as nondiagnostic according to the following criteria.

1. If the teeth, including the periodontal ligament (PDL), could not be seen.
2. If pathology could not be excluded by the radiograph.
3. If other radiographs were needed (especially intraoral) to obtain maximum information.

For every nondiagnostic radiograph, the problematic area was noted as follows.

1. Lower incisors were blurred/shortened/unclear.
2. Upper incisors were blurred/shortened/unclear.
3. Upper and lower incisors were blurred/shortened/unclear.
4. The whole radiograph or large parts of it were blurred/unclear.

Each nondiagnostic radiograph was recorded as a radiograph which needed the addition of periapical radiographs, or to perform a new panoramic radiograph in order to improve its diagnostic power.

### 2.1. Statistical Analysis

Data was processed using SPSS (statistical package for the social sciences) 15.0 software (SPSS Inc., Chicago, IL., USA). Students *t*-test, Fishers exact test, Pearson chi-Square and two-way anova tests were used for the statistical analyses.

## 3. Results

One hundred and forty-three and 146 radiographs of mixed and permanent dentitions, respectively, were examined. The mean age of the patients was 10.55 ± 2.08 and  37 ± 17.65  years, respectively (the ± symbol represents standard deviation (SD)). In the mixed dentition group, there were 57.3% males and 42.7% females. In the permanent dentition group, there were 47.3% males and 52.7% females.

Overall, there were 168 diagnostic and 121 nondiagnostic radiographs.

In each (the mixed and the permanent dentition) group there were only 2 error-free radiographs.

Table 1 shows the mean number of errors in each group. In the mixed dentition group, the mean number of errors per radiograph was significantly lower than in the permanent dentition radiographs (1.74 ± 0.73 and 2.19 ± 0.87, resp., *P* = 0.001). This trend was observed in the diagnostic and nondiagnostic radiographs in each group.

**Figure 1:**
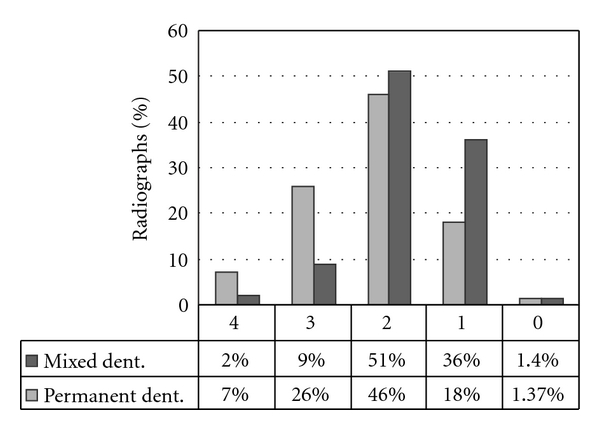
The distribution of errors among the mixed and the permanent dentition groups.

Figure 1 shows the mean number of errors per radiograph in each group. One or two errors were more prevalent in the mixed dentition group; however 3 and 4 errors were more prevalent in the permanent dentition group.

Table 2 shows the frequency of the specific errors in each group. Positioning the patient too far forward was significantly more prevalent in the mixed dentition group (*P* = 0.002), while a slumped position and nonpositioning of the chin on the rest were significantly more prevalent among the permanent dentition group (*P* = 0.001).

Table 3 shows the frequency of errors in the diagnostic and non-diagnostic radiographs in each group. There was no significant difference in the diagnostic and nondiagnostic radiographs in both groups. There were 79 (55%) and 89 (61%) diagnostic radiographs in the mixed and permanent dentition groups, respectively, and 64 (45%) and 57 (39%) nondiagnostic radiographs in the mixed and permanent groups, respectively.

Having the patient positioned too far forward was significantly more prevalent in the diagnostic radiographs in the mixed dentition group, compared to the permanent dentition group (34% and 9%, resp., *P* = 0.04).

Table 4 shows the reasons for problems in the diagnostic power of radiographs. Blurred or shortened upper incisors were significantly more prevalent in the mixed dentition group compared with the permanent group (33.3% and 10.5%, resp., *P* = 0.004).

Table 5 shows the ways to improve the diagnostic power of the panoramic radiographs. The adding of a periapical radiograph of the upper anterior region was significantly more required in the mixed dentition group compared with the permanent dentition group (*P* = 0.01).

In 42% of the radiographs of the mixed dentition group and in 45% of the radiographs of the permanent dentition group, the diagnostic ability could be improved by manipulating the brightness or contrast.

Examining the association between the type of error and the diagnostics of the radiographs revealed the following.

**In the Mixed Dentition Group**

“The patient is positioned too far forward” was associated with 23% of the nondiagnostic radiographs (*P* = 0.003, Pearsons correlation). “The patient is positioned too far backward” was associated with 75%, of the nondiagnostic radiographs (*P* = 0.001, Pearson correlation). “Chin up in the chin rest” was associated with 65.6% of the nondiagnostic radiographs (*P* = 0.009, Pearson correlation).

**In the Permanent Dentition Group**

“The patient is positioned too far back” was associated with 58.3% of the non-diagnostic radiographs (*P* = 0.01). “Slumped position” was associated with 61.8% of the nondiagnostic radiographs (*P* = 0.003).

Tables 6 and 7 show the associations between types of errors and the problems in diagnostic radiographs in the mixed and permanent dentitions, respectively.

In the mixed dentition, tilting the chin down in the rest made the lower incisors to be significantly nondiagnostic (92.9% of the lower incisors were blurred, shorteneds or unclear, *P* = 0.001). In addition, a slumped position resulted in 100% of the lower incisors to be nondiagnostic (blurred, shortened, or unclear, *P* = 0.029).

In the permanent dentition, tilting the chin down in the rest resulted in the lower incisors to be significantly nondiagnostic (72.7% of the lower incisors were blurred, shortened, or unclear, *P* = 0.015). No technical errors were noted.

## 4. Discussion

Our study investigated for the first time and to the best of our knowledge the differences between digital panoramic radiographs of patients with mixed dentition and patients with permanent dentition. Until today, all studies have concentrated on patients with permanent dentition. We used digital radiographs due to their increasing popularity among dentists. The results of our study demonstrate a high frequency of errors in all radiographs examined. All errors originated from improper positioning of the patients but none due to technical errors. This is in agreement with previous studies [15, 16]. One study found that the most common error was that the tongue was not in contact with the hard palate [18].

It would be assumed that errors in panoramic radiographs would have been more prevalent among the younger group (patients with mixed dentition). They may not be calm and motionless during the radiograph procedure (13–20 seconds) and there may be difficulties in properly positioning the children due to the small dimensions of their heads and different body proportions, compared to adults. However, the average number of errors was higher among the older patients with permanent dentitions.

A close look at the distribution of errors in the results of our study reveals that few errors (up to two errors) were more prevalent among the permanent dentition group. However, three and more errors were prevalent among the mixed dentition group.

As for the type of error in the mixed and permanent dentition groups, “the position of the patient is too far forward” was more prevalent among the younger group. This error may originate from the smaller size of the head in the younger age group and the consequent attempt to position them forward in the machine.

The “chin is not positioned on the rest” and the “slumped position” were more prevalent among the permanent dentition group. In the “slumped position”, the patient's head is stretched forward and thus, the neck and the back are not on the same plane. These errors may originate from physical characteristics of the patients, such as a short or thick neck, surplus weight, or when they are very tall [5].

Only two radiographs in each group contained no error. This striking finding is in accordance with previous studies [15, 16, 18]. The explanation for the many errors must be considered in the understanding of the panoramic radiography. The limited dimensions of the focal plane (image layer) in panoramic radiography mean that minor errors in positioning manifest as distortions due to unequal vertical and horizontal magnification, overlap of teeth, and a loss of image sharpness [3, 5, 19].

A most common error in each group was that the tongue was not placed in the proper position. This finding is in agreement with previous studies [5, 16, 18]. When the tongue is misplaced it may create a radiolucent “band” projected on the apices of the maxillary teeth, and thus, diminish the diagnostic ability in this region. It has been claimed that false diagnosis of periapical lesions and cysts may occur [5].

Nearly 40% of the radiographs in our study were nondiagnostic. The criteria for diagnosis in our study were subjective, and the reasons for the panoramic radiographs were unknown to the authors. Because of the dissociation between the evaluation of the radiographs and the clinical condition as well as the reasons for the radiographs, the issue of diagnostic power may not reflect a real problem—for example, a radiograph, whose incisor area is blurred and unclear yet whose area of the third molar, for which the dentist asked the radiograph appears clear [10, 15].

Properly positioning the patient in the machine is the most important factor in preventing a cascade of errors, as multiple mistakes may follow automatically from the first mistake. For example, positioning the chin too low usually results in the patient also being in a slumped position [5]. Equally important is the need for regular monitoring of images made to identify recurring errors and to suggest methods to remedy these errors. In light of our findings, efforts are now made to put special attention to proper positioning of all patients when taking panoramic radiographs. Technicians are constantly instructed about the consequences of errors when taking panoramic radiographs.

The least accurate areas for proper diagnosis in the panoramic radiographs in our study were the upper and lower anterior regions. This is in accordance with the common agreement on the topic [17]. Therefore, complementary periapical radiographs of the anterior region are required.

Our study faces some limitations: the sample sizes were relatively small, and all images have been evaluated by one examiner. Needed are studies on a larger sample, with a variety of examiners, to enhance the strength of the results. Nevertheless, our findings point out an important issue when taking a panoramic radiograph, the proper positioning of the patient.

## Figures and Tables

**Table 1:** Mean number of errors in each group (the ± symbol represents SD).

	Mean number of errors per radiograph	Mean number of errors in diagnostic radiographs	Mean number of errors in nondiagnostic radiographs
Mixed dentition	1.74 ± 0.73	1.61 ± 0.67	1.9 ± 0.8
Permanent dentition	2.19 ± 0.87*	2.04 ± 0.87**	2.4 ± 0.8***

*P < 0.001  *t*-test.

**P < 0.001 two-way anova.

**Table 2:** Frequency of errors in the mixed and the permanent dentition groups*.

Error	Mixed dentition (%)	Permanent dentition (%)	*P*
Pt. too far forward	24.5	10.3	0.002**
Pt. too far back	16.8	24.7	0.112
Head twisted	0.7	4.8	0.067
Head tilted	12.6	14.4	0.732
Chin tipped too low	16.1	15.8	1.000
Chin raised too high	22.4	19.2	0.563
Slumped position	2.1	23.3	0.001**
Chin not on chin rest	11.9	47.9	0.001**
Tongue not on plate	60.1	52.7	0.236
Bite guide not used	0	0.7	1.000
Pt. moves	2.1	1.4	0.682
Pt. wears jewelry	5.6	3.4	0.409

*The following errors were excluded since they did not appear in both groups: no home base, use of lead apron which fits intraoral radiographs or thyroid protector, and Cassette resistance.

**Fisher exact test.

**Table 3:** Frequency of errors in the diagnostic and nondiagnostic radiographs*.

Error	Mixed dentition		Permanent dentition	
	Diagnostic *N* = 79 (55%)	Nondiagnostic *N* = 64 (45%)	Diagnostic *N* = 89 (61%)	Nondiagnostic *N* = 57 (39%)
Pt. too far forward	34.0%	12.5%	9%	12.3%**
Pt. too far back	7.6%	28.1%	16.9%	36.8%
Head twisted	0	1.6%	3.4%	7/0%
Head tilted	12.7%	12.5%	14.6%	14.0%
Chin tipped too low	11.4%	21.9%	13.5%	19.3%
Chin raised too high	13.9%	32.8%	20.2%	17.5%
Slumped position	0	4.7%	14.6%	36.8%
Chin not on chin rest	11.4%	12.5%	48.3%	47.4%
Tongue not on plate	64.6%	54.7%	56.2%	47.4%
Bite guide not used	0	0	1.3%	0
Pt. moves	0	4.7%	1.1%	1.8%
Pt. wears jewelry	5.0%	6.3%	3.4%	3.5%

*The following errors were excluded since they did not appear in both groups: no home base, use of lead apron which fits intraoral radiographs or thyroid protector, and cassette resistance.

**P = 0.04, chi-Square test.

**Table 4:** Reasons for problems in diagnostic power of radiographs.

Reason for nondiagnosis	Mixed dentition	Permanent dentition	P*
Lower incisors blurred/shortened	31.7%	38.6%	0.45
Upper incisors blurred/shortened	33.3%	10.5%	0.004
Lower and upper incisors blurred/shortened	23.8%	31.6%	0.41
Entire radiograph of large parts blurred/unclear	11.2%	19.3%	0.31

*Fisher exact test.

**Table 5:** Ways of improving the diagnostic power of radiographs.

How to improve diagnostics	Mixed dentition	Permanent dentition	P*
Add lower PA	19.8%	21.9%	0.74
Add upper PA	17.1%	5.7%	0.01
Add upper and lower PA	13.5%	17.1%	0.57
Add PA as needed	7.2%	10.5%	0.47

*Fisher exact test.

**Table 6:** The association between the type of error and the problem in the radiograph's diagnostics in mixed dentition.

Type of error	Reason	Percent of nondiagnostic radiographs	P*
Head tilted	Blurred/unclear radiograph	62.5%	0.001
Chin down in rest	Lower incisors blurred/shortened/unclear	92.9%	0.001
Chin down in rest	Upper incisors blurred/shortened/unclear	0	0.003
Chin up in rest	Lower incisors blurred/shortened/unclear	0	0.001
Chin up in rest	Upper incisors blurred/shortened/unclear	75.0%	0.001
Slumped position	Lower incisors blurred/shortened/unclear	100%	0.029

*Fisher exact test.

**Table 7:** The association between the type of error and the problem in the radiograph diagnosis in the permanent dentition*.

Type of error	Reason	Percent of non-diagnostic radiographs	P*
Chin up in rest	Lower incisors blurred/shortened/unclear	72.7%	0.015
Chin down in rest	Lower incisors blurred/shortened/unclear	0	0.005
Chin up in rest	Upper incisors blurred/shortened/unclear	50.0%	0.001
Slumped position	Blurred/unclear radiograph	4.8%	0.041

*Fisher exact test.
